# Single-centre experience with autosomal recessive limb-girdle muscular dystrophy: case series and literature review

**DOI:** 10.1055/s-0043-1772833

**Published:** 2023-10-18

**Authors:** Paulo José Lorenzoni, Cláudia Suemi Kamoi Kay, Renata Dal-Pra Ducci, Otto Jesus Hernandez Fustes, Paula Raquel do Vale Pascoal Rodrigues, Nyvia Milicio Coblinski Hrysay, Raquel Cristina Arndt, Lineu Cesar Werneck, Rosana Herminia Scola

**Affiliations:** 1 Universidade Federal do Paraná, Hospital de Clínicas, Departamento de Clínica Médica, Serviço de Neurologia, Serviço de Doenças Neuromusculares, Curitiba PR, Brazil.

**Keywords:** Muscular Dystrophies, Limb-Girdle, Muscular Diseases, Biopsy, Genetics, Distrofia Muscular do Cíngulo dos Membros, Doenças Musculares, Biópsia, Genética

## Abstract

Limb-girdle muscular dystrophy (LGMD) is a group of myopathies that lead to progressive muscle weakness, predominantly involving the shoulder and pelvic girdles; it has a heterogeneous genetic etiology, with variation in the prevalence of subtypes according to the ethnic backgrounds and geographic origins of the populations. The aim of the present study was to analyze a series of patients with autosomal recessive LGMD (LGMD-R) to contribute to a better characterization of the disease and to find the relative proportion of the different subtypes in a Southern Brazil cohort. The sample population consisted of 36 patients with LGMD-R. A 9-gene targeted next-generation sequencing panel revealed variants in 23 patients with LGMD (64%), and it identified calpainopathy (LGMD-R1) in 26%, dysferlinopathy (LGMD-R2) in 26%, sarcoglycanopathies (LGMD-R3–R5) in 13%, telethoninopathy (LGMD-R7) in 18%, dystroglicanopathy (LGMD-R9) in 13%, and anoctaminopathy (LGMD-R12) in 4% of the patients. In these 23 patients with LGMD, there were 27 different disease-related variants in the *ANO5* , *CAPN3* , *DYSF* , *FKRP* , *SGCA* , *SGCB* , *SGCG* , and *TCAP* genes. There were different causal variants in different exons of these genes, except for the *TCAP* gene, for which all patients carried the p.Gln53* variant, and the *FKRP* gene, which showed recurrence of the p.Leu276Ile variant. We analyzed the phenotypic, genotypic and muscle immunohistochemical features of this Southern Brazilian cohort.

## Resumo

A distrofia muscular de cinturas (DMC) é um grupo de miopatias que leva à fraqueza muscular progressiva, e envolvendo predominante as cinturas escapular e pélvica. A DMCtem uma etiologia genética heterogênea, com variação na prevalência de subtipos de acordo com as origens étnicas e geográficas das populações. O objetivo deste estudo foi analisar uma série de pacientes com DMC do tipo autossômico recessivo (DMC-R) para contribuir para uma melhor caracterização da doença e encontrar a proporção relativa dos diferentes subtipos em uma coorte do Sul do Brasil. A população amostral foi composta por 36 pacientes com DMC-R. O painel de sequenciamento de nova geração com 9 genes revelou variantes em 23 pacientes com DMC (64%), e identificou calpainopatia (DMC-R1) em 26%, disferlinopatia (DMC-R2) em 26%, sarcoglicanopatias (DMC-R3–R5) em 13%, teletoninopatia (D-MCR7) em 18%, distroglicanopatia (D-MCR9) em 13%, e anoctaminopatia (DMC-R12) em 4% dos pacientes. Nesses 23 pacientes com DMC, havia 27 variantes diferentes nos genes *ANO5* , *CAPN3* , *DYSF* , *FKRP* , *SGCA* , *SGCB* , *SGCG* e *TCAP* . Foram encontradas diferentes variantes em diferentes éxons desses genes, com exceção do gene *TCAP* , para o qual todos os pacientes eram portadores da variante p.Gln53*, e do gene *FKRP* , que apresentou recorrência da variante p.Leu276Ile. As características fenotípicas, genotípicas e imuno-histoquímicas musculares desta coorte do Sul do Brasil foram analisadas.

## INTRODUCTION

Limb-girdle muscular dystrophy (LGMD), a group of myopathies with onset after two years of life that primarily affects skeletal muscle, 1 2 3 4 5 6 7 leads to progressive muscle weakness, predominantly involving the shoulder and pelvic girdles, caused by a loss of muscle fibers; 1 2 3 4 5 6 7 it has a heterogeneous genetic etiology, and more than 30 directly-related genes have been reported. 2 4 5 6 8 9 10 Based on the inheritance pattern, LGMD is classified into two major categories: LGMD-D, with autosomal dominant inheritance, and LGMD-R, with autosomal recessive inheritance. 2 3 4 5 6 7 10 The recessive type (previously named LGMD-2) is the most common worldwide, especially calpain3-related LGMD-R1 (MIM#11420), dysferlin-related LGMD-R2 (MIM#603009), α-sarcoglycan-related LGMD-R3 (MIM#600119), β-sarcoglycan-related LGMD-R4 (MIM#600900), γ-sarcoglycan-related LGMD-R5 (MIM#608896), δ-sarcoglycan-related LGMD-R6 (MIM#601411), fukutin-related protein (FKRP)-related LGMD-R9 (MIM#606596), and anoctamin-5( *ANO5* )-related LGMD-R12 (MIM#608662). 1 3 4 5 6 10 11 12 13 14

The clinical heterogeneity of LGMD is generally associated with high molecular variability. 3 14 The spectrum of clinical, laboratory, and radiological features of the LGMD subtypes is broad, but symptoms of limb-girdle muscle weakness associated with increased levels of serum creatine kinase (CK) and fatty substitution of the muscles on magnetic resonance imaging (MRI) scans can suggest the disease. 4 5 6 Therefore, the diagnosis of LGMD may be confirmed by muscle biopsy or genetic studies identifying pathogenic variants in the related genes. 1 4 15 Besides a rehabilitation program, no treatments for LGMD are currently available, despite several ongoing clinical trials. 6 7 8 16 However, accurate diagnosis of LGMD-R is important for patients and their families, to provide genetic counselling and efficient and cost-effective use of medical resources. 7

Studies have described the specific clinical, laboratory, radiological, histological, and genetic features of LGMD-R in series of Brazilian patients. 1 11 12 17 The aim of the present study was to analyze a Southern Brazilian cohort with LGMD-R to contribute to a better characterization of the disease and to evaluate the relative proportion of the different subtypes.

## METHODS

We selected all cases catalogued as LGMD associated with high levels of CK in the tertiary referral center for neuromuscular disorders at Complexo do Hospital de Clínicas of Universidade Federal do Paraná (CHC-UFPR, in the city of Curitiba, Brazil) between 2018 and 2019. Subjects with other neuromuscular disorders (such as, myotonic dystrophy, facioscapulohumeral dystrophy, Duchenne and Becker muscular dystrophy, among others) were excluded. A retrospective analysis of the clinical, laboratory, electrophysiological, radiological and histological features was performed. All studies were conducted in accordance with ethical principles after obtaining from the patients informed consent for muscle biopsies and genetic tests in the outpatient clinic or during hospital admission for diagnostic investigation. The study was approved by the Ethics Committee of CHC-UFPR.

### Clinical evaluation

Relevant data, including age at onset, gender, parental consanguinity, family recurrence, symptoms, and course of the disease were collected. Muscle strength was measured by manual muscle testing (and graded from 0 to 5), according to the Medical Research Council (MRC). Clinical severity at the last appointment was classified according to the MRC sumscore (MRC-SS), 18 which was defined as the sum of scores of the MRC scale for muscle strength from 6 muscles in the upper and lower limbs on both sides, which ranges from 60 (normal strength) to 0 (complete paralysis).

### Laboratory analysis

The highest serum CK level was recorded (normal level: < 168 U/L).

### Electrophysiological indings

The needle electromyography (EMG) pattern, performed at the time of the first evaluation, was classified as normal, myopathic, neurogenic or mixed (myopathic and neurogenic or ‘neurogenic-like’ findings), according to standard procedures. The motor and sensitive nerve conduction studies (NCS) were performed according to standard procedures.

### Radiological findings

The muscle MRI pattern was classified as normal or dystrophic (characterized by fatty substitution of the muscles on imaging) according to standard procedures.

### Muscle biopsy analysis

Muscle biopsies were performed by open surgical procedure on the vastus lateralis or biceps brachialis muscles. The muscle biopsy samples were frozen in liquid nitrogen. Freshly frozen cryostat sections were stained according to standard procedures. 19 The muscle specimens were analyzed by morphological study and immunofluorescence/immunohistochemistry (IF/IHC) for expression analysis, which involved a panel using antibodies against the C-terminus of dystrophin, the rod domain of dystrophin, the N-terminus of dystrophin, dysferlin, α-sarcoglycan, β-sarcoglycan, γ-sarcoglycan, δ-sarcoglycan, calpain-3, and telethonin (commercial antibodies; specification under request). The results of the IF/IHC were classified according to four grades: total, partial, mosaic or no apparent deficiency. The muscle biopsies were performed when genetic testing was not available or was inconclusive, as well as when differential diagnosis with other myopathies was necessary.

### Molecular analysis

We extracted DNA from dried blood spot or blood samples. A targeted next-generation sequencing (NGS) panel was performed (commercial test; specification under request). The panel includes the genes *ANO5* , *CAPN3* , *DYSF* , *FKRP* , *SGCA* , *SGCB* , *SGCD* , *SGCG* , and *TCAP* , based on the reported disease frequency in Latin America and Brazil. 11 12 The variants (such as pathogenic, probably pathogenic or variant of uncertain significance) were classified according to the guidelines of the American College of Medical Genetics and Genomics (ACMG). Sequence variations were compared with data available in the genetic databases (Human Gene Mutation Database [HGMD], Leiden Muscular Dystrophy Database, and ClinVar).

## RESULTS

The sample population consisted of 36 (19 female and 17 male) patients from unrelated families aged between 17 and 71 (median: 39; mean ± standard deviation [SD]: 40.14 ± 14.23) years at disease diagnosis. The age at onset varied between 2 and 59 years, with a median of 18 years and mean ± SD of 21.66 ± 14.51 years. The disease duration varied between 3 and 52 (median: 15; mean ± SD: 18.47 ± 11.92) years. Table 1 summarizes the clinical data of the patients with LGMD-R subtypes.

**Table 1:** Summarized clinical data of the studied patients with autosomal recessive limb-girdle muscular dystrophy (LGMD-R)

LGMD subtype		R1(2A)	R2 (2B)	R3–5 (2CDE)	R7 (2G)	R9 (2I)	R12 (2L)
**Gene**		*CAPN3*	*DYSF*	*SGCA-B-G*	*TCAP*	*FKRP*	*ANO5*
**Patients (n)**		6	6	3	4	3	1
Affected family member (n)*		3	3	3	1	2	0
**Sex (male:female)**		2:4	1:5	3:0	3:1	1:2	1:0
Age at onset *(years)**		24.16 ± 10.83	25.16 ± 11.61	7.33 ± 4.04	8.75 ± 3.50	24.33 ± 12.89	29
Disease duration (years)**		18.17 ± 11.41	21 ± 9.67	24.67 ± 11.55	14.25 ± 8.61	22.33 ± 26.08	15
Medical Research Council sumscore**		42.66 ± 9.09	40.33 ± 15.16	42.66 ± 11.01	45.75 ± 13.57	47.33 ± 3.05	14
**Clinical manifestation**	Cramps Myalgia Asymmetric weakness Shoulder weakness Pelvic weakness Anterior leg weakness Posterior leg weakness Hand weakness Scapular atrophy Shoulder atrophy Scapular winging Pectoral atrophy Hip atrophy Thigh atrophy Leg atrophy Calf hypertrophy Lordosis Scoliosis Joint/tendon contractures Gowers maneuver Myopathic gait	0 0 0 6 6 0 0 0 5 5 4 1 3 3 0 4 1 0 0 5 5	2 2 1 6 4 4 2 1 4 4 2 0 1 1 0 1 1 0 0 2 2	0 0 1 3 3 0 0 0 0 3 3 1 2 2 1 2 2 0 1 1 1	2 2 1 2 4 2 1 0 2 2 3 0 3 3 2 2 2 1 2 2 1	1 1 0 3 3 0 0 0 2 2 2 0 2 2 0 3 2 1 0 3 2	0 0 0 1 1 0 0 0 0 1 0 0 0 1 0 0 1 0 0 0 0
**Wheelchair-bound**		1	2	2	1	0	1
**Cardiac involvement**		1	0	1	0	1	0
**Respiratory involvement**		0	1	0	0	0	0
**Previous misdiagnosis of inflammatory myopathy**		1	3	0	0	1	1
Maximum level fo creatine kinase (U/L)**		3446.33 ± 4789.74	3736.66 ± 3225.61	20641 ± 29782.19	1176.25 ± 468.20	3727.33 ± 3288.13	517
**Needle electromyography (n(**	Normal Myopathic features Mixed features*** Myotonic discharge	0 6 0 0	1 4 1 1	0 3 0 0	0 4 0 0	0 2 1 1	0 1 0 0
**Muscle biopsy features (n)**	Type-1 fiber atrophy Type-2 fiber atrophy Type-1 fiber predominance Hypertrophy of type-1 fiber Hypertrophy of type-2 fiber Bordeline myopathic changes Chronic myopathy Active and chronic myopathy Myopathy with denervation Chronic denervation Lobulated/trabecular fiber Target (core-like) fiber Inflammatory infiltration	0 1 1 0 0 0 3 1 1 0 1 0 0	1 0 0 0 0 0 1 0 2 1 1 1 1	1 0 0 1 1 0 1 1 0 0 0 0 0	0 0 0 0 0 1 2 0 1 0 3 0 0	1 1 0 0 0 0 0 1 0 1 0 0 0	1 0 0 0 0 0 0 0 0 0 0 0 1
**Genetic analysis (n)**	Definitive Probable****	4 2	4 2	2 1	3 1	3 0	0 1

Notes: *Presence of family member with similar clinical characteristics but without definitive diagnosis; **mean ± standard deviation if the calculation was allowed; ***concomitance of myopathic and denervation changes (‘neurogenic-like’); ****only one pathogenic variant (diagnosis supported by other tests, such as histological analyses).

The targeted NGS panel revealed variants in 23 (64%) patients with LGMD, with homozygous variants in 7 patients and compound heterozygous variants in 9 patients. However, the same mutations were also found as a single heterozygous variant in 7 patients. There were 27 different disease-related variants in the *ANO5* , *CAPN3* , *DYSF* , *FKRP* , *SGCA* , *SGCB* , *SGCG* , and *TCAP* genes ( Table 2 ). There were different causal variants in different exons of these genes, except for the *TCAP* gene, for which all patients carried the p.Gln53* variant, and the *FKRP* gene, which showed recurrence of the p.Leu276Ile variant. In this cohort, other affected family members were reported (52%) for almost all subtypes, and parental consanguinity was reported in 5 patients (28%). Previously, 6 genetically-confirmed patients (26%) had been misdiagnosed and treated for polymyositis (1 with calpainopathy, 3 with dysferlinopathy, ‘ with dystroglycanopathy, and 1 with anoctaminopathy).

**Table 2:** Brief summary of the variants found in the studied patients with autosomal recessive limb-girdle muscular dystrophy (LGMD-R) ^#^

Gene	Status ^##^	DNA variant	Protein	Number of alleles
*CAPN3*	Pathogenic	c.328C > T	p.Arg110*	1
*CAPN3*	Pathogenic	c.550del	p.Thr184Argfs*36	1
*CAPN3*	VUS	c.680C > T	p.Ala227Val	1
*CAPN3*	Pathogenic	c.759_761delGAA	p.Lys254del	1
*CAPN3*	Pathogenic	c.1342C > T	p.Arg448Cys	1
*CAPN3*	VUS	c.1775G > A	p.Arg592Gln	1
*CAPN3*	Pathogenic	c.2242C > T	p.Arg748*	1
*CAPN3*	Pathogenic	c.2243G > A	p.Arg748Gln	2
*CAPN3*	Pathogenic	c.2362_2363delAGinsTCATCT	p.Arg788Serfs*14	1
*DYSF*	Pathogenic	c.265C > T	p.Arg89*	2
*DYSF*	Probably pathogenic	c.1163_1165dupCCG	p.Ala388dup	1
*DYSF*	VUS	c.2801C > T	p.Pro934Leu	1
*DYSF*	Pathogenic	c.2875C > T	p.Arg959Trp	1
*DYSF*	Pathogenic	c.2996G > A	p.Trp999*	1
*DYSF*	Pathogenic	c.3031 + 2T > C	p.(?)	1
*DYSF*	VUS	c.3280T > C	p.Trp1094Arg	1
*DYSF*	Pathogenic	c.3805G > T	p.Glu1269*	2
*SGCA*	VUS	c.350G > A	p.Arg117Gln	1
*SGCB*	Pathogenic	c.622–2A > G	p.(?)	1
*SGCB*	Pathogenic	c.341C > T	p.Ser114Phe	1
*SGCG*	Pathogenic	c.353C > G	p.Ser118*	1
*SGCG*	VUS	c.195 + 5G > A	p.(?)	1
*TCAP*	Pathogenic	c.157C > T	p.Gln53*	7
*FKRP*	Probably pathogenic	c.826C > A	p.Leu276Ile	4
*FKRP*	Pathogenic	c.898G > A	p.Val300Met	1
*FKRP*	Pathogenic	c.899T > C	p.Val300Ala	1
*ANO5*	VUS	c.1747G > A	p.Val583Ile	1

Abbreviation: VUS, variant of uncertain significance.

Notes: ^#^ More data about these variants under request (such as population frequency, in silico prediction, segregation, conservation, among others); ^##^ According ACMD classification.

### Calpainopathy (LGMD-R1/LGMD-2A)

In total, six patients were diagnosed with LGMD-R1 ( Table 1 ). Other affected family members were reported by three patients. Consanguinity was reported by one patient. Previously, one patient had been misdiagnosed and treated for polymyositis.

**Figure 1:**
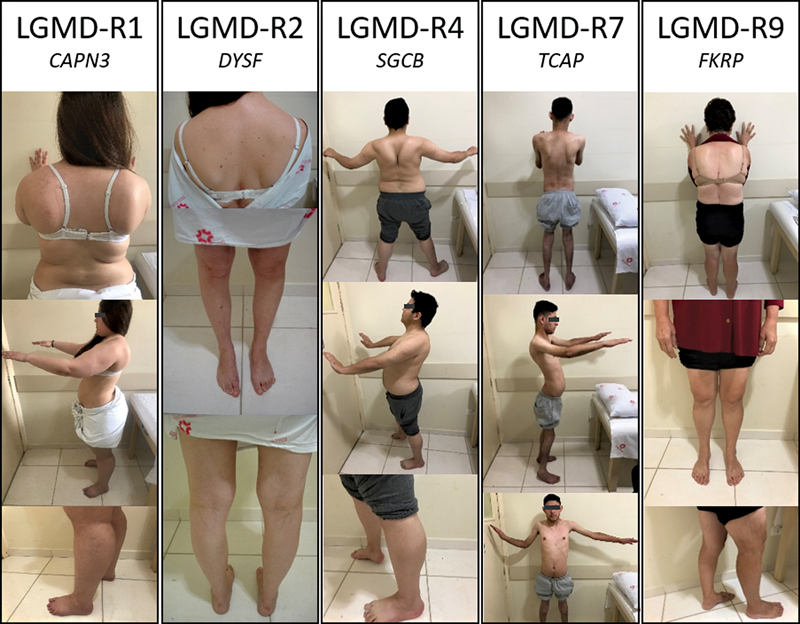
Clinical findings selected from patients with genetically confirmed LGMD-R. Notes: (gene: variants): LGMD-R1 ( *CAPN3* : p.Arg448Cys / p.Arg788Serfs*14); LGMD-R2 ( *DYSF* : p.Trp999* / p.Glu1269*); LGMD-R4 ( *SGCB* : c.622–2A > G[splicing-site] / p.Ser114Phe); LGMD-R7 ( *TCAP* : p.Gln53* / p.Gln53*); and LGMD-R9 ( *FKRP* : p.Leu276Ile / p.Leu276Ile).

Shoulder and pelvic girdle weakness occurred in all patients ( Table 1 ). In the upper limbs, scapular and shoulder atrophy occurred in five patients, scapular wing atrophy, in four patients, and pectoral atrophy, in one patient ( Figure 1 ). In the lower limbs, hip and thigh atrophy

occurred in three patients. Calf hypertrophy was observed in four patients ( Figure 1 ), and one patient had lordosis. Myopathic gait and Gowers maneuver were observed in five patients, and one patient was wheelchair-bound. Cardiac involvement was found in one patient who presented left ventricular hypertrophy on electrocardiography (ECG).

**Figure 2:**
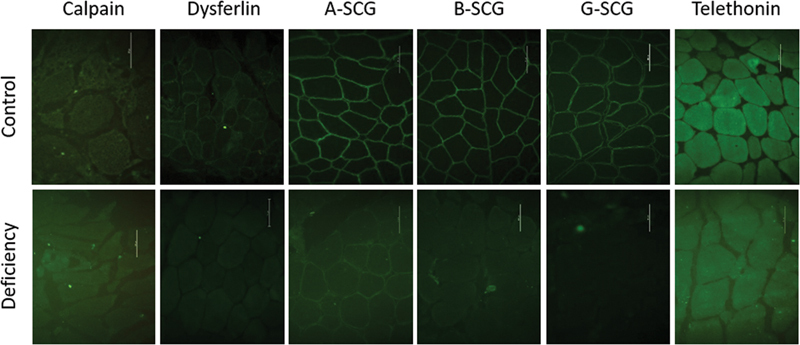
Immunohistochemical analysis of sarcomeric (calpain-3 and telethonin) and sarcolemmal (dysferlin, α-sarcoglycan, β-sarcoglycan, and γ-sarcoglycan) structures showing reduced pattern of the immunofluorescence stain in muscle biopsies of the patients with confirmed LGMD-R compared with controls. Abbreviations: A-SCG, α-sarcoglycan; B-SCG, β-sarcoglycan; G-SCG, γ-sarcoglycan. Note: Bar = 50 µm.

The serum CK levels were elevated in all patients, with the highest level between 536 U/L and 13 thousand U/L ( Table 1 ). The EMG showed myopathic features in all patients, and the NCS showed axonal neuropathy of the left deep peroneal nerve in one patient and demyelinating neuropathy of the bilateral median nerve at the carpal tunnel in another patient, and the remaining patients were normal. Muscle MRI scans were only performed in one patient; they revealed fatty substitution predominantly involving the posterior thigh muscles of the lower limbs. Muscle biopsy was performed in all patients, which disclosed type-2 fiber atrophy associated with type-1 fiber predominance in one patient, concomitant features of myopathy and denervation in one patient, chronic myopathy in two patients, chronic myopathy with lobulated muscle fiber in one patient, and active and chronic myopathy in another patient. The IF/IHC for calpain in muscle tissue was performed, which confirmed calpain deficiency ( Figure 2 ). Based on the IF/IHC analysis for other sarcolemmal proteins, there was partial deficiency of dysferlin in one patient.

In total, nine different mutations were identified in the *CAPN3* gene ( Table 2 ). Most mutations were found only once; they were homozygous variants in one patient, and compound heterozygous variants in three patients. A single heterozygous variant was found in two patients (muscle tissue from both was subjected to IF/IHC; the results confirmed calpain deficiency). Our cohort with LGMD-R1 had four patients with a *definitive* diagnosis and two patients with a *probable* diagnosis ( Table 1 ), who had one single variant associated with calpain deficiency on the muscle biopsy.

### Dysferlinopathy (LGMD-R2/LGMD-2B)

In total, six patients were diagnosed with LGMD-R2 ( Table 1 ). Other affected family members were reported by three patients. Consanguinity was reported by two patients. Previously, three patients had been misdiagnosed and treated for polymyositis.

Cramps occurred in two patients. Two patients had myalgia. In proximal muscles, weakness occurred in the shoulder girdle in all patients, and in the pelvic girdle in four patients. In distal muscles, weakness occurred in the anterior leg muscles in four patients, in the posterior leg muscles in two patients, and in the hand muscles in one patient. The weakness was asymmetric in one patient. The MRC-SS was between 20 and 59 in these patients ( Table 1 ). In the upper limbs, scapular and shoulder atrophy occurred in four patients and scapular wing atrophy occurred in two ( Figure 1 ) subjects. Atrophy in the lower limbs, hip and thigh occurred in one patient ( Figure 1 ). Calf hypertrophy was observed in one patient, and another subject had lordosis. Myopathic gait and Gowers maneuver were observed in two patients. Two subjects were wheelchair-bound. Respiratory involvement was found in one patient who presented mild restrictive pulmonary capacity based on spirometry.

The serum CK levels were elevated in all patients, with the highest level between 782 U/L and 9,257 U/L ( Table 1 ). The EMG was normal in one patient and showed myopathic features in three patients, myopathic with myotonic discharges in one patient, and myopathic with neurogenic findings in one patient. The NCS were normal in all patients. Muscle MRI scans were performed in two patients, which revealed fatty substitution predominantly involving the anterior and posterior thigh muscles of the lower limbs in both patients. Muscle biopsy was performed in all patients, which disclosed concomitant features of myopathy and denervation in two patients, myopathy with trabecular muscle fibers in one patient, inflammatory myopathy in one patient, and denervation with type-1 atrophy associated with target (core-like) muscle fibers and chronic denervation in one patient. The IF/IHC analysis of muscle tissue was performed, which confirmed dysferlin deficiency in all patients ( Figure 2 ). In two patients, the IF/IHC analysis also revealed partial deficiency of other sarcolemmal proteins (sarcoglicans and dystrophin).

In total, eight different mutations were identified in the *DYSF* gene ( Table 2 ). Most mutations were found only once or twice; they were homozygous variants in one patient and compound heterozygous variants in three patients. A single heterozygous variant was found in two patients (muscle from both were subjected to the IF/IHC analysis, which confirmed dysferlin deficiency). Our cohort of LGMD-R2 had four patients with a *definitive* diagnosis and two patients with a *probable* ( Table 1 ) diagnosis, and they had one single variant associated with dysferlin deficiency on muscle biopsy.

### Sarcoglycanopathies (LGMD-R3–R5/LGMD-2C-E)

In total, three patients were diagnosed with sarcoglycanopathies: LGMD-R3 (2D), LGMD-R4 (2E) and LGMD-R5 (2C) were identified in one patient each ( Table 1 ).

The patient with LGMD-R3 was a male in whom the disease had started at 11 years of age, and he had had the disease for 27 years. Other affected family members were reported. The weakness reported in the shoulder and pelvic girdle m uscles were mildly asymmetric. Scapular winging (asymmetric) and shoulder, pectoral (asymmetric), and leg atrophy were observed. The EMG showed myopathic features. The NCS showed axonal neuropathy of the right common peroneal nerve. Muscle biopsy revealed atrophy of type-1 muscle fiber and hypertrophy of type-1 and -2 muscle fibers. The IF/IHC of muscle tissue revealed partial deficiency in α-sarcoglycan ( Figure 2 ). A single heterozygous variant was found in the *SGCA* gene.

The patient with LGMD-R4 was a male in whom the disease had started at 8 years of age, and he had had the disease for 10 years. Other affected family members were reported. The weakness was in the shoulder and pelvic girdle muscles. Scapular winging and atrophy of muscles of the shoulder, pelvic and thigh were observed, as well as lordosis, calf hypertrophy, and joint and tendon contractures ( Figure 1 ). Myopathic gait and Gowers maneuver were observed at onset. The patient was wheelchair-bound most of the time, and had cardiac involvement (heart failure). The EMG showed myopathic features, and the NCS was normal. Muscle biopsy revealed active and chronic myopathy. The IF/IHC of muscle tissue revealed deficiency in β- and γ-sarcoglycan ( Figure 2 ). The variants in the *SGCB* gene were compound heterozygous.

The patient with LGMD-R5 was a male in whom the disease had started at 3 years of age, and he had had the disease for 15 years. Other affected family members were reported. The weakness was in the shoulder and pelvic girdle muscles. Scapular winging and atrophy of the muscles of the shoulder, pelvis and thigh were observed. Calf hypertrophy and lordosis were also present. The patient was wheelchair-bound; the EMG showed myopathic features, the NCS was normal, and muscle biopsy revealed chronic myopathy. The IF/IHC of muscle tissue revealed deficiency in γ-sarcoglycan ( Figure 2 ). The variants in the *SGCG* gene were compound heterozygous.

Among the subjects with LGMD-R3–5, two patients had a *definitive* diagnosis and one patient had a *probable* diagnosis (one single variant associated with α-sarcoglycan deficiency on muscle biopsy; Table 1 ).

### Telethoninopathy (LGMD-R7/LGMD-2G)

In total, four patients were diagnosed with LGMD-R7 ( Table 1 ); one patient reported another affected family member, and another reported consanguinity.

Cramps occurred in two patients, and two subjects had myalgia. In the proximal muscles, weakness occurred in the shoulder girdle in two patients, and in the pelvic girdle occurred in all patients. In the distal muscles, weakness occurred in the anterior leg muscles in two patients, and in the posterior leg muscles in one. The weakness was asymmetric in one patient. The MRC-SS varied between 34 and 58 in these patients ( Table 1 ). In the upper limbs, scapular winging and shoulder atrophy occurred in two patients and scapular winging alone occurred in three ( Figure 1 ). In the lower limbs, hip and thigh atrophy occurred in three participants, and leg atrophy occurred in two patients ( Figure 1 ). Calf hypertrophy was observed in two patients. Two patients had lordosis, and one had scoliosis ( Figure 1 ). Joint and tendon contractures occurred in two patients. Myopathic gait was observed in one patient, and Gowers’ maneuver occurred in two patients. One subject was wheelchair-bound.

**Figure 3:**
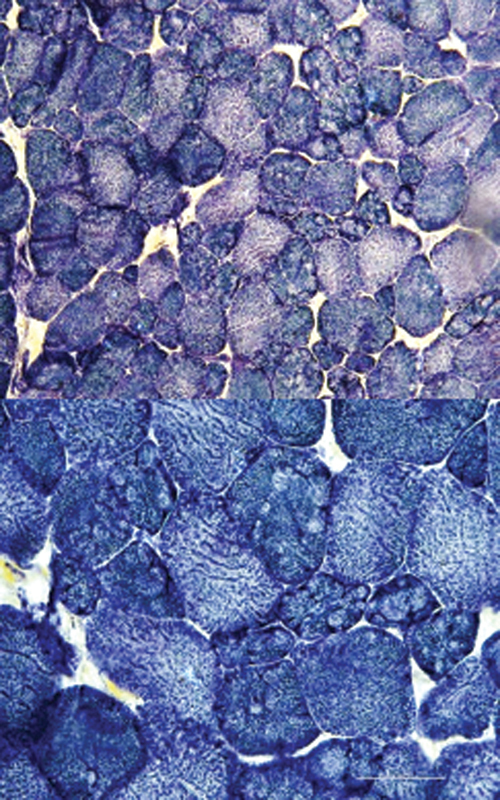
Morphological features of the muscle biopsy showing trabecular (lobulated) fibers in a patient with LGMD-R7 (nicotinamide adenine dinucleotide dehydrogenase stain). Note: Bar = 50 µm.

Serum CK levels were elevated in all patients, with the highest level between 662 U/L and 1,710 U/L ( Table 1 ). The EMG showed myopathic features in all patients, and the NCS was normal. Muscle biopsy was performed in every patient, which disclosed chronic myopathy with trabecular muscle fibers in two patients ( Figure 3 ), concomitant features of myopathy and denervation associated with trabecular muscle fibers in one patient, and borderline to myopathic features (muscle fibers with variation in size and increased acid phosphatase reaction) in one patient. The IF/IHC analysis on muscle tissue was performed, which confirmed telethonin deficiency in all patients ( Figure 2 ).

Only one mutation (p.Gln53*) was identified in the *TCAP* gene ( Table 2 ); it was a homozygous variant in three patients, and as single heterozygous variant in one patient. As for LGMD-R7, three patients had a *definitive* diagnosis, and one patient had a *probable* diagnosis (one single variant associated with teletonin deficiency on muscle biopsy) ( Table 1 ).

### Fukutin-related proteinopathy (LGMD-R9/LGMD-2I)

In total, three patients were diagnosed with LGMD-R9, which is also named dystroglycanopathy by fukutin-related proteinopathy ( Table 1 ). Other affected family members were reported by two patients, and consanguinity was reported by one patient. Previously, one patient had been misdiagnosed and treated for polymyositis, and two patients had undergone spinal surgery to correct scoliosis.

Cramps occurred in one patient, and another patient had myalgia. Weakness occurred in the shoulder and pelvic girdle in all patients. The MRC-SS was between 44 and 50 in these patients ( Table 1 ). In the upper limbs, scapular winging, as well as scapular and shoulder atrophy, occurred in two patients ( Figure 1 ). In the lower limbs, hip and thigh atrophy occurred in two patients ( Figure 1 ). Calf hypertrophy was observed in all patients ( Figure 1 ). Two patients had lordosis, and one had scoliosis ( Figure 1 ). Myopathic gait was observed in two patients, and Gower maneuver occurred in all patients. One patient had cardiac involvement (myocardiopathy).

The serum CK levels were elevated in all patients, with the highest level between 1,800 U/L and 7,524 U/L ( Table 1 ). The EMG showed myopathic features in one patient, myopathic features with myotonic discharges in one patient, and myopathic with neurogenic findings in one patient. The NCS was normal. Muscle MRI was performed in two patients, which revealed fatty substitution involving the thigh muscles of the lower limbs (predominantly posterior) in two patients, and paraspinal muscles in one patient. Muscle biopsy was performed in all patients, which disclosed active and chronic myopathy in one patient, chronic denervation in one patient, and atrophy of type-1 and type-2 muscle fibers in one patient. The IF/IHC of muscle tissue revealed partial deficiency (mosaic pattern) in dysferlin and dystrophin in one patient.

In total, only three mutations were identified in the *FKRP* gene ( Table 2 ); they were homozygous variants in two patients and a compound heterozygous variant in one patient. All patients with LGMD-R9 had a *definitive* diagnosis ( Table 1 ).

### Anoctaminopathy (LGMD-R12/LGMD-2L)

One patient was diagnosed with probable *ANO5* -related LGMD ( Table 1 ). The patient was a male in whom the disease had started at 29 years of age, and he had had it for 15 years. Previously, the patient had been misdiagnosed and treated for polymyositis. Weakness occurred in the shoulder and pelvic girdle muscles. Shoulder and thigh atrophy were observed, as well as lordosis The patient was wheelchair-bound; The EMG showed myopathic features, the NCS was normal, and the muscle biopsy disclosed atrophy of type-1 fibers with inflammatory endomysial infiltration. The IF/IHC analysis of muscle tissue showed partial deficiency of dysferlin. A single heterozygous variant was found in the *ANO5* gene. The patient was classified with a *probable* diagnosis ( Table 1 ).

### Negative group

The targeted NGS panel did not reveal variants related to LGMD in 13 patients (36%) of the cohort, who were called the *negative group* : 7 female and 6 male subjects, from unrelated families, aged between 27 and 71 (median: 40; mean ± SD: 43.38 ± 13.69) years at disease diagnosis. The age at onset varied between 2 and 59 years, with a median of 23 years and mean ± SD of 25 ± 18.46 years. The disease duration varied between 6 and 43 (median: 12; mean ± SD: 18.38 ± 12.75) years. The MRC-SS was between 36 and 60 (median: 48; mean ± SD: 46 ± 7.48) in these patients. The serum CK levels were elevated in 12 patients and normal in 1 patient (median: 495 U/L; mean ± SD: 2,769.84 ± 3,664.89 U/L).

## DISCUSSION

In the present study, there were variations in the prevalence of LGMD subtypes according to the ethnic background and geographic origin of the subjects, as well as based on founder mutations. 3 6 7 In a large American study 8 that employed a 35-gene NGS panel and examined patients with clinical suspicion of LGMD, most subjects had variants in the *CAPN3* (17%), *DYSF* (16%), *FKRP* (9%), and *ANO5* (7%) genes. In 290 participants of the MYO-SEQ project, which is a Europe-wide project using whole exome sequencing (WES) to diagnose patients with unexplained limb girdle weakness from over 50 centres, calpainopathy (77 patients) was the most frequently diagnosed subtype of LGMD, followed by dysferlinopathy (48), anoctaminopathy (31), titinopathy (25), and sarcoglycanopathy (16 patients). 4 In addition, a total of 24 patients of this project were diagnosed with dystroglycanopathies based on pathogenic variants in 5 of the relevant genes ( *FKRP* , *POMT1* , *POMT2* , *FKTN* and *GMPPB* ). 4 A study 15 from Italy found the most common groups were calpainopathy (28.4%), followed by dysferlinopathy (18.7%), sarcoglycanopathies (18.1%), and fukutin-related proteinopathy (6.4%). Another multicentre study 14 confirmed that calpainopathy (30%) and dysferlinopathy (22.6%) are the most frequent subtypes in Italy, followed by sarcoglycanopathies (21.3%), fukutin-related proteinopathy (8.7%), and anoctaminopathy (4.2%). In an Australian cohort, 20 based on the traditional methods of histopathology and candidate gene sequencing, calpainopathy was the most common LGMD-R, followed by dysferlinopathy and fukutin-related proteinopathy, sarcoglycanopathies, anoctaminopathy, and telethoninopathy. However, even though these cohorts have similarities in the frequencies of the LGMD-R subtypes, the prevalence of subtypes varies in different countries. 12 A cohort from the Netherlands 9 was screened by Sanger sequencing; calpainopathy was the most prevalent LGMD-R subtype (28%), but sarcoglycanopathy occurred in 27% of the patients, anoctaminopathy, in 26%, dysferlinopathy, in 10%, and fukutin-related proteinopathy, in 9% of the patients. In Northern England, 13 calpainopathy was the most common (26.5%), followed by fukutin-related proteinopathy (19.1%), sarcoglycanopathy (11.7%), and dysferlinopathy (5.9%). In a Danish cohort, 21 fukutin-related proteinopathy was the most common (36.8%), followed by sarcoglycanopathy (22.3%), calpainopathy (11.6%), and dysferlinopathy (1.9%).

Although recent studies that evaluated LGMD genetics reported a Brazilian profile of LGMD-R, there is great heterogeneity in ethnic backgrounds in Brazil. Hence, the genetic profile could be quite different among regions. 11 In Brazil, calpainopathy and dysferlinopathy are the most frequent subtypes, with rates between 20% and 30%. 1 11 Sarcoglycanopathies are usually the third most frequent subtype, with rates around 20%. 1 11 Telethoninopathy and fukutin-related proteinopathy have similar rates that vary between 5% and 10%. 1 11 Anoctaminopathy has also been ‘emerging’ as a frequent subtype of LGMD-R in Brazil, but with rates around 5%. 11 22 In a recent Latin American study 12 which included Brazilian patients, the researchers found different rates of variants, with dysferlinopathies (39.8%) being the most frequent subtype, followed by calpainopathy (21%), sarcoglycanopathies (13.5%), anoctaminopathy (8.6%), fukutin-related proteinopathy (5.2%), and telethoninopathy (3.5%). Even with the small size of the present study, the frequencies of the most common LGMD-R subtypes reported for our cohort are similar to the those reported for other Brazilian cohorts, mainly from the Southeast and Midwest regions. 11

In a previous analysis performed in our center 17 (based only on the IF/IHC analysis), sarcoglycanopathies were the most frequent LGMD-R subtype (32%), followed by dysferlinopathy (14.3%), and calpainopathy (8.9%), but they occurred in different proportions in the current study. At that time, other studies in Brazil 23 24 25 (mainly with patients from the Southeast region) had reported a higher proportion of sarcoglycanopathies, based on Sanger sequencing and/or IF/IHC analysis, followed by dysferlinopathy and calpainopathy. After that, patients carrying two pathogenic mutations were reported with apparently normal IF/IHC (such as calpain and dysferlin), suggesting that the relative proportion of some subtypes screened through protein analysis might be underestimated. 1 13 15 26 Indeed, the correlation between protein expression based on IF/IHC (and/or western blot) and the probability of identifying mutations in a specific gene has been reported to be higher for sarcoglycans and dysferlin than for calpain. 6 15 We speculate that this phenomenon has contributed to the different frequencies of LGMD-R, which, in the previous studies, had been determined mainly by IF/IHC. In addition, sarcoglycanopathies have been reported as conditions with onset in childhood, usually before 10 years of age, and this LGMD-R subtype has been reported mostly in children (68% of the patients). 2 9 10 In the present study, the age at onset of sarcoglycanopathies and telethoninopathy was younger than for other LGMD-R subtypes, which confirms that the onset of these subtypes usually occurs in childhood. It is also possible that the previous studies included younger patients, a factor that would justify the different proportions of LGMD-R subtypes (such as sarcoglycanopathy).

Notwithstanding these diagnostic difficulties, calpainopathy and dysferlinopathy showed remarkable genetic heterogeneity in our cohort, with nine different mutations in the *CAPN3* gene and eight in the *DYSF* gene ( Table 2 ). This genetic heterogeneity for these subtypes has also been observed worldwide. 14 Interestingly, 18% of our patients had telethoninopathy, which has been considered an ultrarare subtype worldwide. The frequency of this subtype in our cohort was higher than in other Brazilian and international cohorts, including an Italian cohort that found this subtype in only 0.2% of patients. 8 11 12 14 23 This subtype was first described in Brazilian families that carry the homozygous p.Gln53* variant in the *TCAP* gene. 27 This was the only variant in our patients with telethoninopathy ( Table 2 ), a finding that suggests this variant is recurrent in this LGMD-R subtype. Fukutin-related proteinopathy (LGMD-R9) is the most common of the dystroglycanopathies. 3 In the United Kingdom and Denmark, fukutin-related proteinopathy is one of the most frequent subtypes, with rates between 18% and 38%, whereas in North America, Italy and the Netherlands, the rate is between 6.7% and 9%. 8 9 13 15 21 This subtype also has lower rates in Brazil. 11 12 In our cohort, the genetic analysis revealed the recurrence of the p.Leu276Ile variant in the *FKRP* gene. This variant is the most common mutation for this subtype. 1 9 21 28 A recent analysis of clinical and genetic data from the Global FKRP Registry 28 has demonstrated that patients with fukutin-related proteinopathy who are homozygous for the common mutation (p.Leu276Ile) are more likely to have a milder phenotype and later disease onset than patients who are compound heterozygous for the common mutation, or who have a mutation other than the common mutation. Based on our cohort, the *TCAP* and *FKRP* genes have low allelic heterogeneity (based on the number of unique pathogenic variants). Anoctaminopathy-related LGMD-R has been reported as the most common phenotype in Brazilian patients with mutations in the *ANO5* gene, with a mild disease compared with other common subtypes, such as calpainopathy, dysferlinopathy or sarcoglycanopathy. 22 Our patient had only a single variant of uncertain significance and was classified as possibly having anoctaminopathy; however, more studies must be performed to genetically confirm the anoctaminopathy as the cause of his LGMD phenotype.

The serum CK levels can vary during the course of disease. They are usually more elevated in the preclinical or initial stages of the disease, and tend to present a gradual decline according to the progression of the disease, because of the gradual loss of muscle bulk. 4 14 17 The CK increase is mild in telethoninopathy (LGMD-R7), but it is generally higher in others recessive forms. 1 6 11 27 This phenomenon occurred in our cohort: although CK levels were increased in all LGMD-R subtypes, the increase was not as large in the patients with telethoninopathy.

The progressive loss of the muscle fibers results in the generation of short-duration and small-amplitude motor unit potentials with early recruitment in weak muscles seen on EMG. 7 10 14 17 However, neurogenic motor unit potentials can also be observed in areas with clustering or fiber hypertrophy, due to motor unit remodelling caused by segmentary necrosis, which can isolate the distal portions of the muscular fibers from the endplate. 14 29 This might be why ‘neurogenic-like’ motor unit potentials have been described in calpainopathy, dysferlinopathy, and sarcoglycanopathies. 14 17 In our cohort, myopathic motor unit potentials were found often in all LGMD-R subtypes, but this feature was concomitant with myotonic discharges or ‘neurogenic-like’ findings only in some patients with dysferlinopathy and fukutin-related proteinopathy.

The implementation of genetic diagnosis for LGMD has reduced the use of muscle biopsies. However, muscle biopsy is still performed in the investigation of LGMD when the genetic analysis is not available, as functional evidence of pathogenicity for novel variants (usually by IHC for the codified protein) or in the differential diagnosis of other myopathies. The dystrophic changes observed with muscle biopsy, usually characterized by variations in the size of the muscle fibers, necrosis/regeneration, and an increase in the endomysial and perimysial connective tissue, are the landmark of LGMD. 4 14 17 21 30 31 Although the dystrophic changes do not characterize any specific subtype of LGMD-R, some features can provide diagnostic clues. The existence of structural alterations is a nonspecific finding for LGMD-R, but there have been reports 5 17 27 31 32 of the formation of rimmed vacuoles in telethoninopathy and lobulated (trabecular) fibers mainly in calpainopathy and telethoninopathy, among others. Our patients with telethoninopathy presented lobulated muscle fibers. Eosinophilic infiltrates, with or without peripheral blood hypereosinophilia resembling eosinophilic myositis, may be an early and transient feature in calpainopathy. 5 6 Inflammatory reaction on muscle biopsy can be found in some LGMD-R subtypes (such as calpainopathy, dysferlinopathy, fukutin-related proteinopathy, and anoctaminopathy), which contains inflammatory cells and interstitial infiltrates, as observed in our patients with dysferlinopathy and anoctaminopathy. 3 5 6 14 31 32 33 Muscle biopsy may show prominent inflammatory exudates, mimicking an inflammatory myopathy in dysferlinopathy, or it may demonstrate vasculitis in anoctaminopathy, which are both LGMDs with defective membrane repair. 5 Indeed, the patients with dysferlinopathy and anoctaminopathy in our cohort had been misdiagnosed and treated for inflammatory myopathy. In LGMD-R, muscle IHC analyses can be used either as a diagnostic standard or as functional evidence of pathogenicity, mainly for novel or single variants. The IF/IHC is a useful method to phenotypically classify patients with LGMD-R. 14 17 31 Muscle IHC analysis may provide a clear and highly specific result in sarcoglycanopathy, dysferlinopathy and telethoninopathy. 6 15 In dystroglycanopathies, a secondary reduction of laminin-a2 and the glycosylated epitope of α-dystroglycan may be observed on the muscle IF/IHC analysis, reflecting the common pathological feature of these disorders. 6 In calpainopathy, western blot analysis with the presence of abnormal calpain can be useful in the histological diagnosis, mainly in inconclusive cases by IF/IHC or genetic analysis. 1 17 26 31 The western blot analysis can be also helpful in other subtypes, such as dysferlinopathy and telethoninopathy. 1 17 26 31

The diagnosis of LGMD-R has always been challenging. Besides the traditional methods (such as Sanger sequencing and muscle IHC), new methods (like the NGS) have been added to the diagnostic tools. There are several approaches to NGS, including whole genome sequencing (WGS), WES, and multigene target panels. 10 Screening using multigene targeted NGS panels has emerged as the first-line investigation in patients with LGMD-R, but the success rate in the final diagnosis varies greatly among populations. 6 11 12 Although it is difficult to compare the success rate in studies on the diagnosis of LGMD-R that used targeted NGS panel, some studies have reported rates between 10.8% and 59.8% in the identification of mutations using targeted NGS panels in cohorts of patients with LGMD-R. 3 6 8 12 14 34 35 That difficulty is due to the difference in the inclusion criteria and/or in the number of genes included in the panel profile. The 9-gene targeted NGS panel used in the present study identified variants in 64% of the patients. In comparison, a Latin American study and an Argentinian study that used a similar targeted NGS panel identified mutations in 55.8% and 10.8% of the cohort respectively. 12 34

To date, several distinct mutations that cause LGMD-R have been described in the genes; they can be found in different databases, such as ExAC, ClinVar, HGMD and LOVD. However, sometimes the mutant alleles cannot be identified by NGS. Among the 36 patients in our cohort, 23 had genetically-confirmed LGMD-R, 7 (30%) of whom with only a single mutation, diagnosed in the *CAPN3* , *DYSF* , *SCGA, TCAP* , and *ANO5* genes. Therefore, even through genetic analysis is the gold standard for the diagnosis of calpainopathy, previous reports 13 26 32 have demonstrated that only a single pathogenic mutation is identified in ∼ 10% to 16% of the patients with calpainopathy, many *CAPN3* mutations located in a deep intronic position, for example, that could be missed during routine gene sequencing. In this situation, other diagnostics tools (such as multiplex ligation-dependent probe amplification[MLPA] and muscle IF/IHC) could be helpful, especially to identify a second allele defect, when heterozygous variants were initially detected by NGS analysis and muscle biopsy has dystrophic changes. 6 Indeed, in some of our patients with single variants, the diagnosis was also based on the analysis of protein expression by muscle IHC. The MLPA method has been reported to help in the work-up of LGMD-R, for screening whole-exon deletions/duplications, but other genetic methods could be helpful as well, mainly when only a single variant is found on NGS. 9 Our patients with single variants did not undergo other genetic analyses (such as MLPA). As diagnostic tools for LGMD-R, both protein analysis and genetic tests are complex and require careful interpretation in light of the clinical findings.

The small size of the cohort has limited the present study. However, some authors discuss that this is also a strong point, because it represents the real-world frequency of LGMD-R subtypes in our region, and it contributes to better understand LGMD-R in Brazil.

In conclusion, these results are of great importance to understand the epidemiology of LGMD-R in Brazil. The present study has shown that the frequency of LGMD-R subtypes in Southern Brazil is similar to what has been reported in Southeastern and Midwestern Brazil as well as North America, it but differs from that found in Latin America and some European countries. 9 13
